# miR-502 medaited histone methyltransferase *SET8* expression is associated with outcome of esophageal squamous cell carcinoma

**DOI:** 10.1038/srep32921

**Published:** 2016-09-08

**Authors:** Cuiju Wang, Jianhua Wu, Yue Zhao, Zhanjun Guo

**Affiliations:** 1 Department of Gynaecology Ultrasound, The Fourth Hospital of Hebei Medical University, Shijiazhuang, P. R. China; 2 Animal Center, The Fourth Hospital of Hebei Medical University, Shijiazhuang, P. R. China; 3 Department of Gastroenterology and Hepatology, The Fourth Hospital of Hebei Medical University, Shijiazhuang, P. R. China

## Abstract

The histone methyltransferase *SET8,* whose expression is regulated by miR-502 though the binding site in the 3′ UTR of SET8, implicated in cancer development. Single nucleotide polymorphism (SNP) of rs16917496 located in the miR-502 and SET8 binding site was analyzed in esophageal squamous cell carcinoma (ESCC) patients, the *SET8* C/C genotype was independently associated with longer post-operative survival by multivariate analysis (relative risk, 2.250; 95% CI, 1.041–4.857; *p* = 0.039). Moreover, the reduced SET8 expression mediated by *SET8* C/C genotype was associated with longer ESCC survival. Functional assay indicated that the *SET8* knock down could inhibit proliferation and promote apoptosis of ESCC cells. The subsequent assay also showed the markedly inhibition of ESCC cell migration and invasion by *SET8* knock down. Our data suggested that the altering SET8 expression, which is mediated at least partly by miR-502 through changing the binding affinity between miR-502 and *SET8* 3′ UTR, could modify the ESCC outcome by inhibiting the proliferation and invasion as well as promoting the apoptosis of ECSS cell. Our data indicated that SET8 was a new target for ESCC therapy.

Esophageal cancer is responsible for 406800 deaths in 2008, which made it the fifth cancer related deaths in men and the eighth in women worldwide^1^. Esophageal cancer with esophageal squamous cell carcinoma (ESCC) as the major pathological type is one of the most commonest cancers in the population from northern central China with an age-standardized annual incidence rate >125/100,000^1^,^2^. Despite the improved diagnosis and therapeutic strategies, the prognosis of esophageal cancer remains poor mostly due to its aggressive nature. The clinical characteristics including the performance status, the TNM stage and lymph node metastases seem to be the predictive factors for esophageal cancer outcome; moreover, some molecular factors such as p53 mutaion and NF-kappaB expression level also show predictive power for esophageal cancer outcome^3^,^4^.

MicroRNAs (miRNAs) are ~22 nucleotides RNA molecules that act as posttranscriptional regulators of mRNA expression by base pairing to the “seed region” of 2–8 nucleotides in the 3′ untranslated region (UTR) of mRNAs^5^,^6^. The perfect complementarity between the miRNA and its target mRNA sequence results in reduced protein levels due to RNA silencing^7^,^8^. Increasing evidences indicated that single nucleotide polymorphisms (SNPs) in the 3′ UTR alter the targeted genes’ expression and thereby affect an individual’s cancer risk^9^,^10^. PR-Set7/Set8/KMT5a (SET8), which is regulated by miR-502 though the binding site in the 3′ UTR of *SET8* mRNA, encodes a histone H4 lysine 20 monomethyltransferase that is implicated in normal cell cycle progression^11^,^12^,^13^. Human PrSet7 interacts directly with the DNA replication factor PCNA and shows specific effects at origins of replication, *SET8* depletion causes cells to accumulate in S phase with increased DNA damage, moreover, inappropriate SET8 expression also causes S phase defects and increased DNA damage^14^,^15^,^16^,^17^. Recent studies showed that *SET8* activation was essential for p53BP1 recruitment during DNA double-strands break response, furthermore, SET8 could promote epithelial-mesenchymal transition and confer TWIST dual transcriptional activities thereby to increased metastasis capacity of breast cancer cells^18^,^19^.

In our previous studies, we found the rs16917496 SNP including C/C, C/T and T/T genotypes in the “seed” region of *SET8* 3′ UTR where miR-502 binding was associated with both cancer risk of epithelial ovarian cancer and outcome of hepatocellular carcinoma, small cell lung cancer and non-Hodgkin’s lymphomas^20^,^21^,^22^,^23^. In this study, we genotyped this SNP in ESCC patients in a case-control study to assess its relationships to cancer risk and outcome.

## Results

### *SET8* genotype is associated with ESCC survival

A total of 180 ESCC patients were enrolled in this study, the postoperative survival of these patients according to the clinical characteristics were analysed with log-rank test using the Kaplan–Meier method, the Clinical stage was identified for its association for ESCC survival by univariate analysis (Table 1).

**Figure 1:**
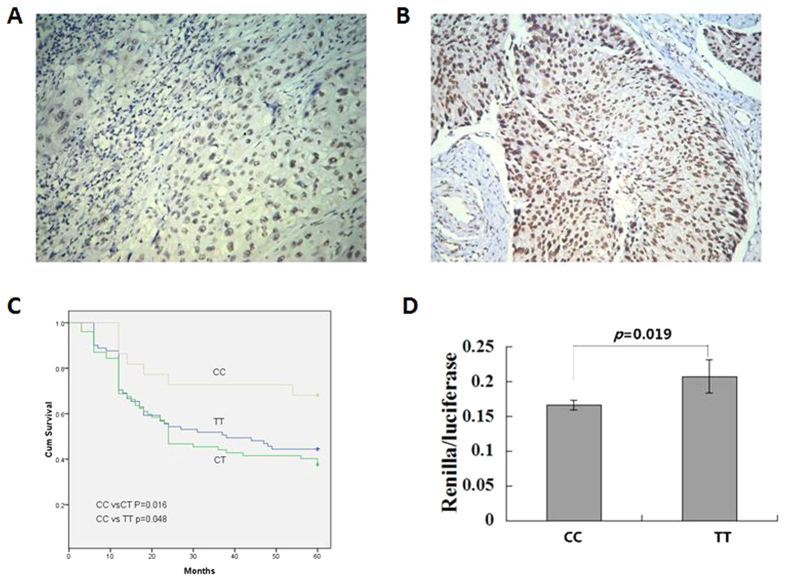
The rs16917496 SNP was associated with survival and SET8 expression in ESCC patients. (**A**) low SET8 expression in ESCC tissues, original magnification: ×200; (**B**) high SET8 expression in ESCC tissue, original magnification: ×200; (**C**) significant survival difference of ESCC patients among C/C, C/T and T/T genotype of rs16917496; (**D**) Renilla luciferase assay for TT and CC genotypes of rs16917496 in Eca109 cell lines. The Renilla luciferase activities is defined as the ratio of Renilla luciferase activities versus firefly luciferase activities.

The *SET8* genotype based on rs16917496 SNP were genotyped in 180 ESCC patients and 142 controls, the distribution of *SET8* genotype followed a Hardy-Weinberg equilibrium. The *SET8* C/C, C/T and T/T genotype frequencies in controls was comparative to genotype frequency in ESCC patients, no difference for SET8 genotype distribution frequency between ESCC and controls had been found (data not shown). The ESCC patients were divided into three groups on the basis of their *SET8* C/C, C/T and T/T genotype for Kaplan–Meier analysis, the 5 years survival rate of patients were 68.2% for C/C type, 37.7% for C/T and 44.4% for T/T, respectively (Fig. 1C). The patients with C/C allele was associated with a significantly longer survival time compared with that of C/T and T/T patients (C/C versus C/T, *p* = 0.016; C/C versus T/T, *p* = 0.048).

The ESCC patients were divided into two groups as CC versus C/T + T/T *SET8* genotypes for further multivariate analysis with the Cox proportional hazards model (Table 2). After adjusted with other ESCC survival associated predictor, the SET8 genotype was identified as an independent predictor for ESCC outcome (relative risk, 2.250; 95% CI, 1.041–4.857; *p* = 0.039).

### miR-502 mediate SET8 expression through their binding site

Because miR-502 biding to the “seed region” of *SET8* 3′ UTR, we evaluate the biological relevance of their biding site SNP of rs16917496 on SET8 expression. SET8 expression was immunostained in 67 ESCC tissues and the HSCORE was calculated. The allele based SET8 expression in ESCC patients are listed in Table 3. We found that the SET8 C allele, which might have perfect complementarity in the seed sequence for miR-502 binding, was associated with lower levels of SET8 expression than that of the T allele by χ^2^ test (*p < *0.001). After survival analysis with Kplan-Meier method, we found that the patients with the low SET8 expression displayed longer survival length than that of high SET8 patients (five years survival rate, 85.7% vs. 30.0%, *p* = 0.022). We evaluate the binding affinity between miR-502 and SET8 genotype with Renilla/luciferase reporter assays, as shown in Fig. 1D, a vector containing the C/C or T/T genotype of *SET8* was constructed in the 3′ UTR of the Renilla luciferasegene and transfected into Eca109 cell line. Consistent with immnostainig results in ESCC tissue, a dramatic reduction of Renilla luciferase activity was observed in C/C genotype of *SET8*. These results indicate that the rs16917496 SNP in the 3′ UTR of *SET8* changed the binding affinity between miR-502 and *SET8* so as to affect SET8 expression.

### *SET8* knock down inhibits proliferation, induces apoptosis, suppresses migration and invasion of ESCC cells

**Figure 2:**
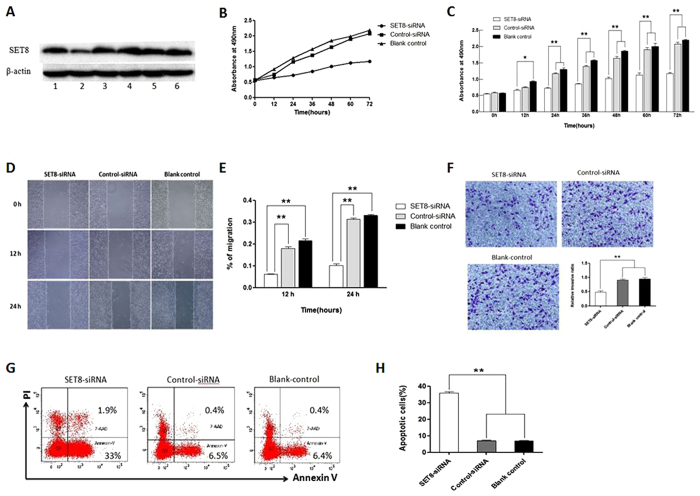
*SET8* knockdown inhibits proliferation, induces apoptosis, suppresses migration and invasion of ESCC cells. (**A**) Western blot analysis with anti-SET8 and anti-β-actin antibodies to selection of SET8siRNA in Eca109 cells; (**B**) *SET8* knockdown inhibits ESCC cell proliferation by MTT assay; (**C**) Quantification of results of B; (**D**) *SET8* knockdown suppresses migration of ESCC cells by wound healing assay; (**E**) Quantification of results of D; (**F**) *SET8* knockdown suppresses invasion of ESCC cells by transwell assay; (**G**) *SET8* knockdown induces apoptosis of ESCC cells by flow cytometry analysis; (**H**) Quantification of results of G. **p ≤ 0.01; *p ≤ 0.05.

Eca109 cells were successfully transfected four psi-H1-*SET8siRNAs*, as shown in Fig. 2A, the *SET8*siRNA2 ligated in psi-H1 plasmid could reduce the protein levels of SET8 dramatically in comparing with other SET8siRNAs, so we use *SET8*siRNA2 for further analysis.

MTT assay was performed to measure proliferation capacity of Eca109 cells with three different treatments (psi-H1-*SET8siRNA*, psi-H1 and blank control), the proliferation rate of Eca109 cells was significantly decreased from 24 to 72 hr after psi-H1-*SET8siRNA* transfection compared with that of psi-H1 and blank control group (*p* < 0.01, Fig. 2B,C). To further investigate the mechanism of the cell proliferation inhibition by SET8, apoptosis was measured by Annexin V-PE/7-AAD double staining with flow cytometry. Apoptotic cells were significantly augmented in psi-H1-*SET8siRNA* group compared with that of the control vector and blank control groups (*p* < 0.01, Fig. 2G,H). These results indicated that knockdown of SET8 could inhibit proliferation and induce apoptosis of Eca109 cells.

We evaluate the effects of *SET8* knockdown on cell migration and invasion with wound healing assay and transwell assay subsequently, as shown in Fig. 2D,E, Eca109 cells transfected with psi-H1-*SET8siRNA* displayed significantly decreased migration capacity than that of control vector and blank control by wound healing assay (*p* < 0.05). Invasion capacity was assessed by transwell assay, the invasive ratio was dramatically decreased in psi-H1-*SET8siRNA* transfected ESCC cells than that in psi-H1 transfected ESCC cells and blank control (Fig. 2F, *p* < 0.01). These results indicated that SET8 knock down could inhibit the migration and invasion of Eca109 cells.

**Figure 3:**
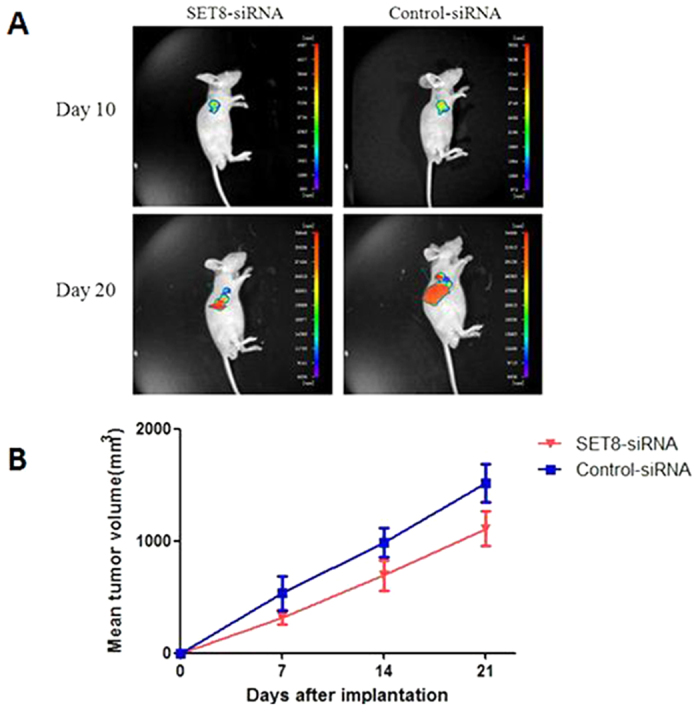
SET8 knockdown inhibited the growth of ESCC cell *in vivo*. (**A**) fluorescence image of mice bearing human Eca109 xenografts. (**B**) Tumor growth was monitored by measuring the mean tumor volume (*p* < 0.01, SET8-siRNA group vs. control-siRNA group at 21 day time point after implantation).

The growth rate of xenograft consisted with SET8-siRNA stably transfected Eca109 cells was compared with xenograft consisted with control-siRNA stably transfected Eca109 cells. As shown in Fig. 3, the growth of SET8-siRNA xenograft was significantly decreased compared with that of control-siRNA xenograft, the tumor volume of SET8-siRNA xenograft was smaller than that of control-siRNA xenograft at the time point of 21 days after implantation (Fig. 3B, *p* < 0.01). These data demonstrated that the SET8 knockdown could inhibit ESCC cell growth *in vivo*.

## Discussion

The mechanism of miR-502 mediated SET8 expression and their implication in ESCC development were assessed in this study. We showed firstly that the rs16917496 SNP in the miR-502 binding site of the *SET8* 3′ UTR was associated with ESCC survival by multivariate analysis; secondly we showed that the SNP resulting in the T to C transition might destroy the G:C bond in miR-502 and *SET8* binding site so as to modulate SET8 expression by immunostaining and luciferase reporter assays; thirdly we showed that SET8 expression was associated with ESCC outcome; finally we showed that the reduced *SET8* could inhibit proliferation and invasion as well as increase apotosis for ESCC cell of Eca109. We also assessed the role of SET8 knockdown on ESCC cell of TE1 referring to proliferation, invasion and apoptosis, the same results was obtained as it do in Eca109 cells (data not shown). The expressional level of miR-502 was measured in ESCC tissue and Eca109 cells, there is no expressional difference for miR-502 among ESCC tissue of CC, CT and TT genotype, the miR-502 expressed moderately and the rs16917496 SNP belong to TT type in Eca109 cells (data not shown). Our data suggest that the altering SET8 expression, which is mediated at least partly by miR-502, could modify the ESCC outcome by inhibiting the proliferation and invasion as well as promoting the apoptosis of ESCC cell. SET8 knockdown also was found to inhibit ESCC cell growth *in vivo.*

A number of SNPs within the microRNA binding site were identified for their associations with cancer risk and outcome^22^,^24^,^25^,^26^,^27^. The rs16917496 SNP in the miR-502 and *SET8* biding site were identified for their relationships to outcome of hepatocellular carcinoma, small cell lung cancer and non-Hodgkin’s lymphomas with C/C genotype associating to longer survival in our previous study^20^,^21^,^22^,^23^, which is comparable to our data in ESCC. Consistent with our and Song’s data demonstrating that the C/C genotype of *SET8* is associated with low expression in breast cancer and hepatocellular carcinoma tissue, we show that the C/C genotype is associated with low protein level and longer survival length of ESCC patients^22^,^28^. These data demonstrated the general mechanism of miR-502 mediated SET8 expression in modifying cancer development.

As a methyltransferase, SET8 methylates lysine 382 of p53 to modulates p53 activity so as to change its transcriptional activity for downstream targets, furthermore, *SET8* knockdown has been shown to upregulate cells’ sensitivity to cell death and cell cycle arrest following DNA damage by suppressing the biological function of p53^29^. *SET8* could promote the epithelial–mesenchymal transition (EMT) and enhance the invasion potential of breast cancer cell mostly by methylating the promoters of the TWIST target genes E-cadherin and N-cadherin via its H4K20 monomethylation activity^30^. Set8-Numb-p53 signaling axis is an important regulatory pathway for apoptosis and SET8 could methylate Numb to abolish its apoptotic function in breast cancer cells^18^. Consistent with previous study, we found that SET8 konckdown could inhibit proliferation and invasion as well as increase apoptosis of ESCC. Furthermore, *SET8* is functionally required for 53BP1 recruitment during DNA double-strand breaks (DSBs), depletion of *SET8* could abrogate 53BP1’s accumulation at DSBs so as to make cell sensitive for apoptosis^19^. The true mechanism of *SET8* in ESCC process and their relationships to p53 activity need further deduce.

Our results indicated that SNPs of microRNA binding site have an effect on cancer outcome, but the results from this study require validation in other populations and in laboratory-based functional studies. SET8 could modify the cancer outcome through their effect on proliferation and invasion, therefore, SET8 would be a new therapy target for ESCC treatment.

## Methods

### Tissue specimens and DNA extraction

Blood samples were collected at the Fourth Hospital of Hebei University from 180 ESCC patients who underwent tumor resection in the Department of Surgery between 2004 and 2008. Blood was also collected from age-matched healthy controls. Genomic DNA was extracted immediately with a Wizard Genomic DNA extraction kit (Promega, Madison, WI). All procedures were supervised and approved by the hospital’s Human Tissue Research Committee. Inform consent were provide to all patients enrolled in this study and the methods were carried out in accordance with the approved guidelines.

### PCR amplification and sequence analysis

The DNA fragments flanking rs16917496 in the *SET8* 3′ UTR was amplified with the forward primer 5′- TCACGACGGTGCTACCTAAG-3′ and reverse primers 5′- CATGCTGGTGTGACACAGTC-3′according to the NCBI database (http://www.ncbi.nlm.nih.gov/snp/) using a PCR Master Mix Kit (Promega). Cyclic sequencing was performed with a Dye Terminator Cycle Sequencing Ready Reaction Kit (Life Technologies, Carlsbad, CA) and separated using an ABI PRISM Genetic Analyzer 3100 (Life Technologies). Polymorphisms were confirmed by repeating the analysis on both strands.

### Measurement of SET8 levels in ESCC tissue

The ESCC tissue immunostaining was performed with an anti-SET8 antibody (Abcam, Cambridge, UK) at a dilution of 1:100 overnight at 4 °C, followed by incubation with a biotinylated secondary anti-mouse IgG antibody for one hour at room temperature. HRP-conjugated streptavidin was used for incubation with the ESCC tissue section subsequently and 3, 3-diaminobenzidine (DAB) was used for staining developing.

The stained slides were semi-quantified using the HSCORE as described previously by two pathologist who were blinded to the sequencing data^22^. Briefly, the percentage of positively-stained ESCC cells in each of 5 intensity categories (0, 1+, 2+, 3+ and 4+) was calculated. The HSCORE represents the sum of each of the percentages multiplied by the weighted intensity of staining as follows: HSCORE = (i + 1)π, where i = 1, 2, 3 and 4 and π varies from 0 to 100%. High expression defined as a score of >100% and low expression defined as a score <100 (Fig. 1A,B).

### Renilla/luciferase reporter assays

Four oligonucleotides, containing from 5′ to 3′: XhoI stick end (5 bp), a fragment from the 3′ untranslated region (UTR) of *SET8 *gene containing the C/C or T/T genotype (rs16917496; 52 bp), and an NotI sticky end (2 bp), were synthesized, sense for CC genotype (5′-TCGAGGTTTGTGGTTTAGCTTTGTATTTAAACAAGGAAATAAACTTGAAAATTATTTGC -3′) and antisense for CC genotype (5′-GGCCGCAAATAATTTTCAAGTTTATTTCCTTGTTTAAATACAAAGCTAAACCACAAACC-3′); sense for TT genotype (5′-TCGAGGTTTGTGGTTTAGCTTTGTATTTAAATAAGGAAATAAACTTGAAAATTATTTGC-3′); and antisense for TT genotype (5′-GGCCGCAAATAATTTTCAAGTTTATTTCCTTATTTAAATACAAAGCTAAACCACAAACC-3′). The four oligonucleotides were first annealed with 1× NEBuffer 2 (New England Biolabs, Ipswich, MA) in a heating block at 95 °C for 5 minutes, followed by a gradual reduction of temperature to room temperature. The psiCheck2 vector (Promega) containing Renilla luciferase and controlled firefly luciferase genes was linearized by digestion with NotI and XhoI (New England Biolabs) and purified from an agarose gel. The annealed oligonucleotides were ligated in the linearized psiCheck2 vector into the NotI and XhoI cloning sites located downstream of the Renilla luciferase reporter gene with T4 DNA ligase (Promega). The ligated vectors were transformed in Escherichia coli competent cells, and positive clones were selected by sequencing.

The human esophageal carcinoma cell line Eca109, purchased from the Institute of Biochemistry and Cell Biology, Chinese Academy of Sciences (Shanghai, China), were seeded in 48-well plates and cotransfected with has-miR502-5p miRNA mimics and modified psiCheck2 vector containing either the C/C or T/T genotype. The Renilla luciferase activity was measured with a luminometer (Lumat, Albuquerque, NM) 48 hours after transfection with the Dual-Lucy Assay Kit (Vigorous Instrument, Beijing, China), and the transfection efficiency was normalized with the firefly luciferase activities.

### Cell culture and transfection

The Eca109 cells were cultured in RPMI-1640 medium (Gibco^TM^Life Technologies, Grand Island, NY) supplemented with 10% fetal bovine serum(FBS) (Gibco^TM^Life Technologies), 50 IU/ml of penicillin and 50 ng/mL of streptomycin (Gibco^TM^Life Technologies) at 37 °C in a humidified atmosphere containing 5% CO_2_.

Eca109 cells were transfected with psi-H1-*SET8siRNA* and psi-H1 plasmids (GeneCopoeia, Rockville, MD) using Lipofactamine 2000 (Invitrogen, San Diego, CA) according to the manufacturer’s instruction. The following four siRNAs against SET8 termed SET8siRNA1, SET8siRNA2, SET8siRNA3 and SET8siRNA4, the target sequence of the SET8siRNA1 was CAGAAUCGCAAACUUACGGA, the SET8siRNA2 was GAAUGAAGAUUGACCUCAUCG, the SET8siRNA3 was GCCUAGGAAGACUGAUCAAU, and the SET8siRNA4 was GGCGCUCACUGAAGUGUAUG, respectively. Successful knockdown of SET8 was confirmed by western blotting analysis with anti-SET8 antibody (Abcam).

### Cell proliferation assay

Cell proliferation was analysed by methyl thiazolyl tetrazolium (MTT, Sigma, CA) assay as described previously^31^. Cells were seeded in a 96-well microplates with a density of 1 × 10^3^cells per well and incubate for 0–72 hours after *SET8*-siRNA transfection, 20 μl (5 mg/ml) of MTT diluted in PBS was added into every wells and incubated for 4 hours with 5% humidified CO_2_ at 37 °C. The cells were then dissolved with 200 μl of dimethylsulphoxide and the absorbance of the cells was measured by a MicroplateReader (Bio-Rad, Hercules, CA) at 490 nm.

### Wound-healing assay

Eca109 cells were cultured in 6-well culture plates and transfected with psi-H1-*SET8siRNA* for 48 hours. When the cells were cultured to almost 100% confluence, a 200 μl pipette tip scratched across the center of the well and the wounds were recorded at 0 hours, 6 hours, 12 hours, and 24 hours with Image J of version 1.42q (NIH). At least five fields were analysed for each scratch and the percentage of migration was calculated as the width of a scratch divided by the initial width of the same scratch^32^.

### Cell invasion assay

*In vitro* cell invasion assay was performed with transwell permeable membrane with 8-μm pore size in 24-well plate (Corning, NY) as described previously^33^. After transfection with psi-H1-*SET8siRNA* for 48 hours, Eca109 cells (1 × 10^4^) suspended in 100 μl serum-free RPMI-1640 medium were filled onto the upper chamber coated with 1 mg/mL Matrigel (BD Biosciences, NJ), incubated at 37 °C for 24 hours, then the cells invaded into the underside of the membrane were fixed with 500 mL of methanol for 15 minutes and stained with 0.1% crystal violet. Stained cells were counted using the Image J software, and 5 random fields were counted at 100× magnification.

### Flow cytometry

Cell apoptosis was analyzed by PE Annxin V Apoptosis Detection Kit 1(BD Biosciences Pharmingen, San Diego, CA) as described previously^34^. Cells were seeded in 6-well plate to quantify apoptosis according to manufacturer’s instructions, Each group of cells was collected after transfection for 48 hours, and washed twice with cold PBS and once in Binding Buffer and then stained with PE annexin V and 7-aminoactinomycin D (7-AAD) for 15 minutes in the dark at 25 °C. Cell apoptosis were analysed by flow cytometry with FACS Aria II flow cytometer (BD Biosciences). Each experiments was repeated three times.

### Tumor cell xenograft in nude mice

The experimental protocol for the present animal study was approved by the Institutional Animal Care and Use Committee at The Fourth Hospital of Hebei Medical University. Four-week-old female nude mice of the Balb/c strain were purchased from Charles River Laboratories [Beijing, China; permission no. SCXK (Jing) 2012-0001]. The mice were bred in a clean environment at the Experimental Animal Center in the Fourth Hospital of Hebei Medical University and fed a cobalt-60-irradiated mouse diet. The animal experiments were conducted in accordance with the USA National Institutes of Health guidelines for the care and use of laboratory animals^35^. In total, 5 × 10^6^ SET8-siRNA stably transfected and control-siRNA stably transfected Eca109 cells suspended in 200 μl dimethylsulfoxide (DMSO; Tokyo Chemical Industry, Tokyo, Japan) were subcutaneously inoculated into the left scapular region of the mice. The xenograft growth was monitored and when it reached a size of ~100 mm^3^ at 7 days post-inoculation, the long and short diameters of the tumor were measured using a beam caliper every 7 days and the tumor volume was calculated using the following formula: Tumor size = (π × long diameter × short diameter2)/6.

The green fluorescent images for the xenograft were taken using the NightOwl Bioimager (Berthold Technologies, Bad Wildbad, Germany) once a week. Signal intensity was evaluated using WinLight32 software (Berthold Technologies).

### Statistical analysis

The distribution of expression grades for each *SET8* genotype was compared using a χ^2^ test. Survival curves were calculated using the Kaplan–Meier method, multivariate survival analysis was performed using a Cox proportional hazards model. Renilla/luciferase reporter assays, MTT, migration and invasion assay were compared by the Student’s *t* test. P < 0.05 was defined as statistically significant. Statistical analyses were performed using SPSS using the SPSS 18.0 software package (SPSS Company, Chicago, IL).

## Additional Information

**How to cite this article**: Wang, C. *et al*. miR-502 medaited histone methyltransferase *SET8* expression is associated with outcome of esophageal squamous cell carcinoma. *Sci. Rep.* **6**, 32921; doi: 10.1038/srep32921 (2016).

## Figures and Tables

**Table 1:** Univariate analysis of SET8 genotype and clinical characteristics associated with overall survival in esophageal cancer patients.

Characteristic	Case	5-year survival rate(%)	*P*
Gender			0.192
Male	124	41.9	
Female	56	50.0	
Age(year)			0.917
≤60	69	44.9	
>60	111	44.1	
Size of the tumor(diameter)			0.314
≤5 cm	119	47.1	
>5 cm	61	39.3	
Tumor location			0.890
Upper-part	27	40.7	
Middle-part	122	44.3	
lower-part	31	48.4	
Clinical stages			<0.001
0 + I	36	80.6	
II + III	144	35.4	
SET8 genotype			
T/T	81	44.4	0.048^a^
C/C	22	68.2	0.016^b^
C/T	77	37.7	

^a^C/C Genotype vs. C/T Genotype;

^b^C/C Genotype vs. C/T Genotype.

**Table 2:** Multivariate analysis of prognostic factors associated with overall survival in esophageal squamous cell carcinoma patients with COX proportional hazards model.

Factors	Relative risk	95%CI	*P* value
SET8 genotype	2.250	1.042~4.857	0.039
Clinical stages	4.660	2.157~10.069	<0.001

**Table 3:** The distribution frequency of SET8 expressional levels for each allele by χ2 test.

	Low expression	High expression	*p* value
T	6	46	<0.001
C	12	10	
